# Parent-reported behavioral changes following equine-assisted therapy in children with autism spectrum disorder: a single-group longitudinal study

**DOI:** 10.3389/fpsyg.2026.1869472

**Published:** 2026-06-05

**Authors:** Taner Atasoy, Aydın Pekel, Vesile Şahiner Güler, Mehmet Behzat Turan, Mehmet Soyal, Barış Karaoğlu

**Affiliations:** 1 Faculty of Sport Science, Department of Physical Education and Sports, Istanbul Gelisim University, Istanbul, Türkiye; 2 Faculty of Sport Science, Department of Sports Management, Marmara University, Istanbul, Türkiye; 3 Department of Physical Education and Sports, Institute of Health Sciences, Erciyes University, Kayseri, Türkiye; 4 Faculty of Sport Science, Department of Recreation, Erciyes University, Kayseri, Türkiye; 5 Faculty of Sport Science, Department of Coaching Education, Istanbul Gelisim University, Istanbul, Türkiye; 6 Faculty of Sport Science, Department of Recreation, Bingöl University, Bingöl, Türkiye

**Keywords:** autism behavior checklist, autism spectrum disorder, behavioral functioning, equine-assisted therapy, longitudinal study, parent report

## Abstract

**Background:**

Equine-assisted therapy (EAT) has received growing attention as a complementary approach associated with behavioral outcomes among children with Autism Spectrum Disorder (ASD). Previous studies have reported changes across behavioral and social domains following equine-assisted programs; however, evidence remains limited regarding the persistence of parent-reported behavioral changes over time. Therefore, the present study aimed to examine parent-reported behavioral changes following participation in a structured EAT program among children with ASD.

**Method:**

A total of 36 children with ASD (18 girls, 18 boys; mean age = 9.90 ± 1.44 years) participated in this longitudinal study. A single-group pretest–posttest design with a two-week follow-up assessment was employed. Behavioral outcomes were evaluated using the Autism Behavior Checklist (ABC) through face-to-face parent reports. The intervention consisted of EAT sessions held twice weekly for 8 weeks. Repeated-measures ANOVA was used to examine changes across assessment points.

**Results:**

Significant differences were observed across measurement points in parent-reported ABC subdomains, including sensory-related behaviors, relationship-building, body and object use, social and self-care skills, and language-related behaviors (*p* < 0.01). Total ABC scores decreased from pretest to posttest, and some changes appeared to be partially maintained at follow-up. Effect size estimates suggested moderate-to-large within-group changes across parent-reported behavioral domains.

**Conclusion:**

The findings suggest significant within-group changes in parent-reported ABC scores following participation in the EAT program. However, because physical literacy was not directly measured and the study lacked a control group, the findings should be interpreted as preliminary behavioral change patterns rather than evidence of causal intervention effects or physical literacy development. Further controlled studies with multidimensional assessment approaches are needed to clarify the specificity and long-term patterns of observed behavioral changes.

**Clinical trial registration:**

Identifier [NCT07131436].

## 1 Introduction

Animal-Assisted Interventions (AAI) constitute a broad conceptual framework encompassing structured practices that intentionally integrate animals into therapeutic, educational, or supportive contexts to facilitate human well-being. Within this framework, interventions differ according to the type of animal involved, the practitioner’s professional background, and the targeted outcomes. Equine-Assisted Interventions (EAI) represent a specific category of AAI in which horses are central to the intervention process, enabling both mounted and unmounted activities to pursue psychosocial, behavioral, or functional goals. A more narrowly defined modality within equine-based practices is hippotherapy, which is delivered exclusively by licensed physical, occupational, or speech therapists and employs the horse’s movement as a clinical tool to elicit neuromotor and sensory responses. In contrast, EAT, as adopted in the present study, refers to a structured, goal-oriented therapeutic approach that uses interaction with horses under professional supervision to support behavioral and functional outcomes, without positioning the intervention as a direct clinical rehabilitation method. Clarifying these hierarchical and functional distinctions is essential to ensure conceptual precision and terminological consistency in equine-assisted research (Fine, 2019; Santaniello et al., 2020; Peters et al., 2020).

Unlike interventions targeting isolated developmental domains, EAT involves simultaneous engagement in motor coordination, sensory adaptation, communication, and environmental responsiveness. This multidimensional structure may be particularly relevant for children with ASD, whose participation challenges often emerge across interconnected behavioral and functional domains (Peters et al., 2020; Xiao et al., 2023).

Regular physical activity has been shown to reduce maladaptive behaviors and support attention, emotional regulation, and social skills in individuals with ASD (Karakılçık et al., 2023; Özcan and Yıldırım, 2011). However, maintaining motivation and sustained engagement in conventional physical activity settings remains challenging for many children with ASD. Children with ASD frequently experience co-occurring motor coordination difficulties and atypical sensory processing patterns, which may reduce successful participation in conventional movement environments. Such challenges may contribute to lower engagement in organized physical activities and limit opportunities for positive movement experiences that support psychosocial and behavioral development (Toscano et al., 2018; Huang et al., 2020).

Despite the growing body of literature on EAT, important gaps remain that justify the present study. Recent systematic reviews and meta-analyses have demonstrated promising improvements in social communication, social functioning, irritability, and hyperactivity following equine-assisted interventions for children with ASD (Xiao et al., 2023; Chen et al., 2022). Parent-reported measures may be particularly valuable in autism intervention studies because behavioral improvements observed in structured therapeutic environments do not always generalize to everyday contexts. Caregiver observations can therefore provide ecologically meaningful evidence regarding whether intervention-related changes extend to home, school, and community participation (Xiao et al., 2023).

These gaps highlight the need for studies that combine longitudinal designs with ecologically valid assessment methods and a holistic theoretical framework. In addition, greater emphasis on ecologically relevant behavioral outcomes may help clarify whether intervention-related changes extend beyond structured therapy settings and become reflected in everyday participation contexts for children with ASD (Peters et al., 2020).

**Figure 1:**
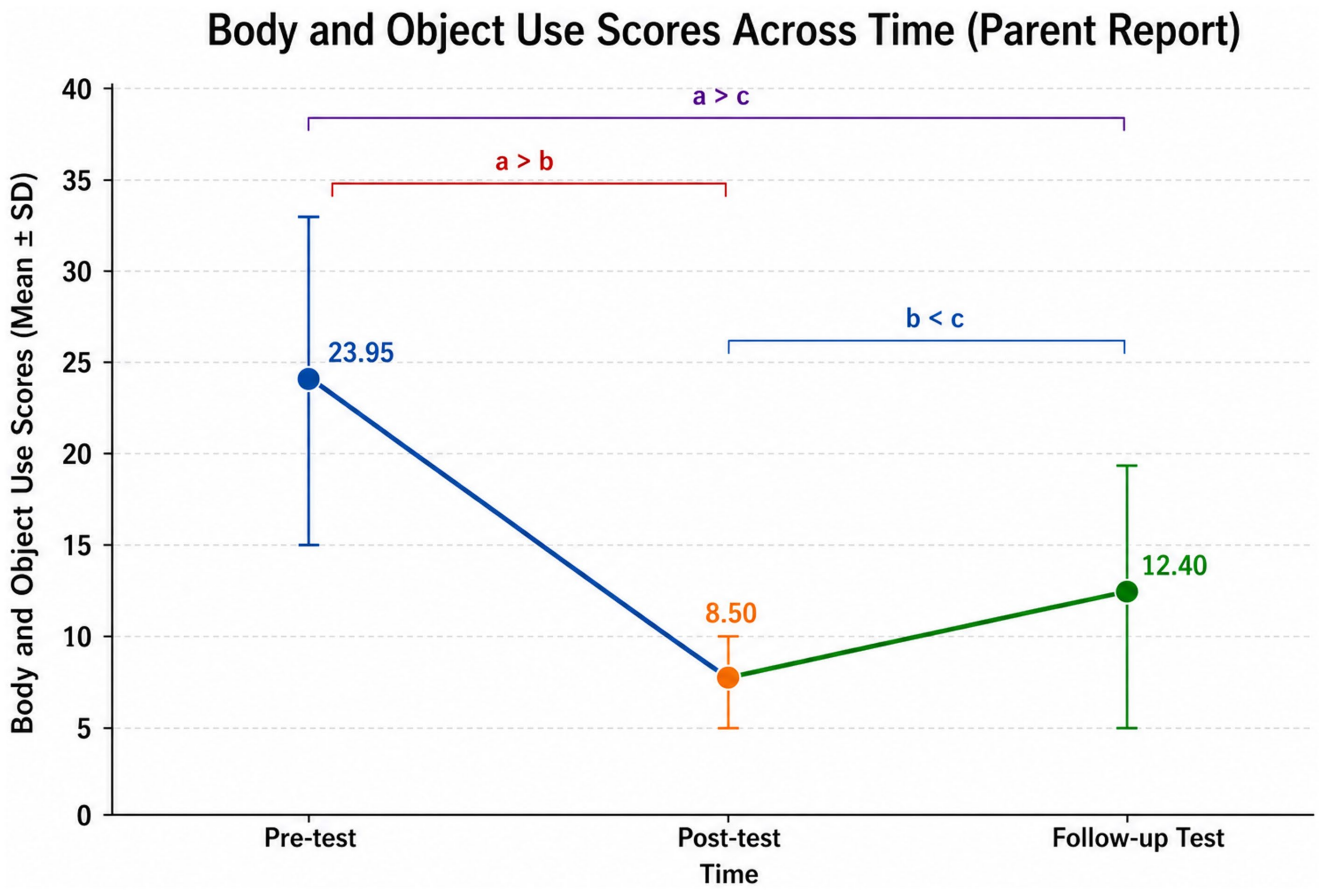
Change in participants’ body and object use scores over time (Main Report).

**Figure 2:**
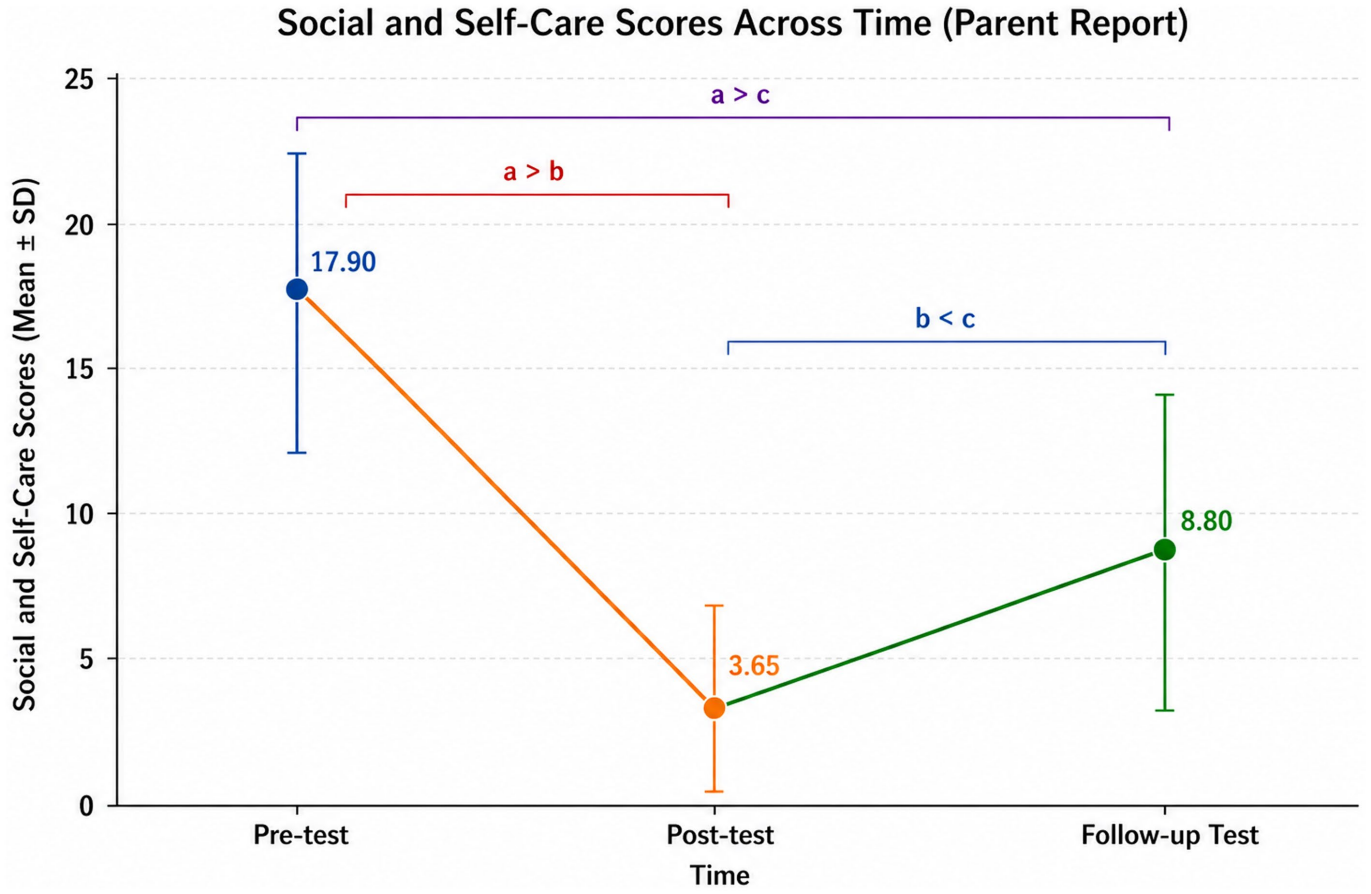
Social and self-care scores over time (Parent Report).

**Figure 3:**
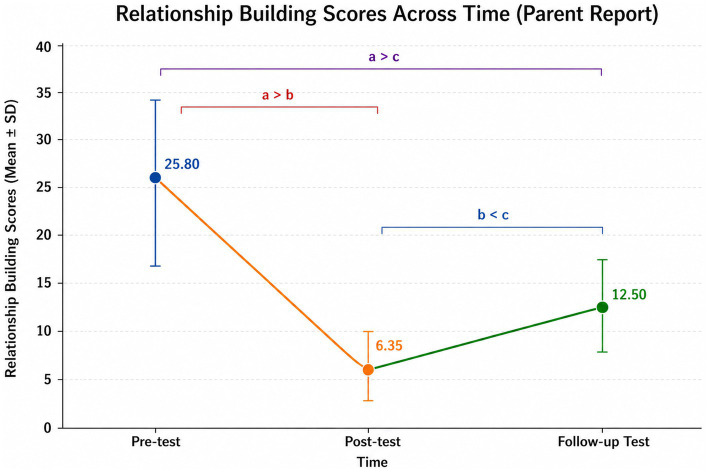
Relationship building scores over time (Parent Report).

**Figure 4:**
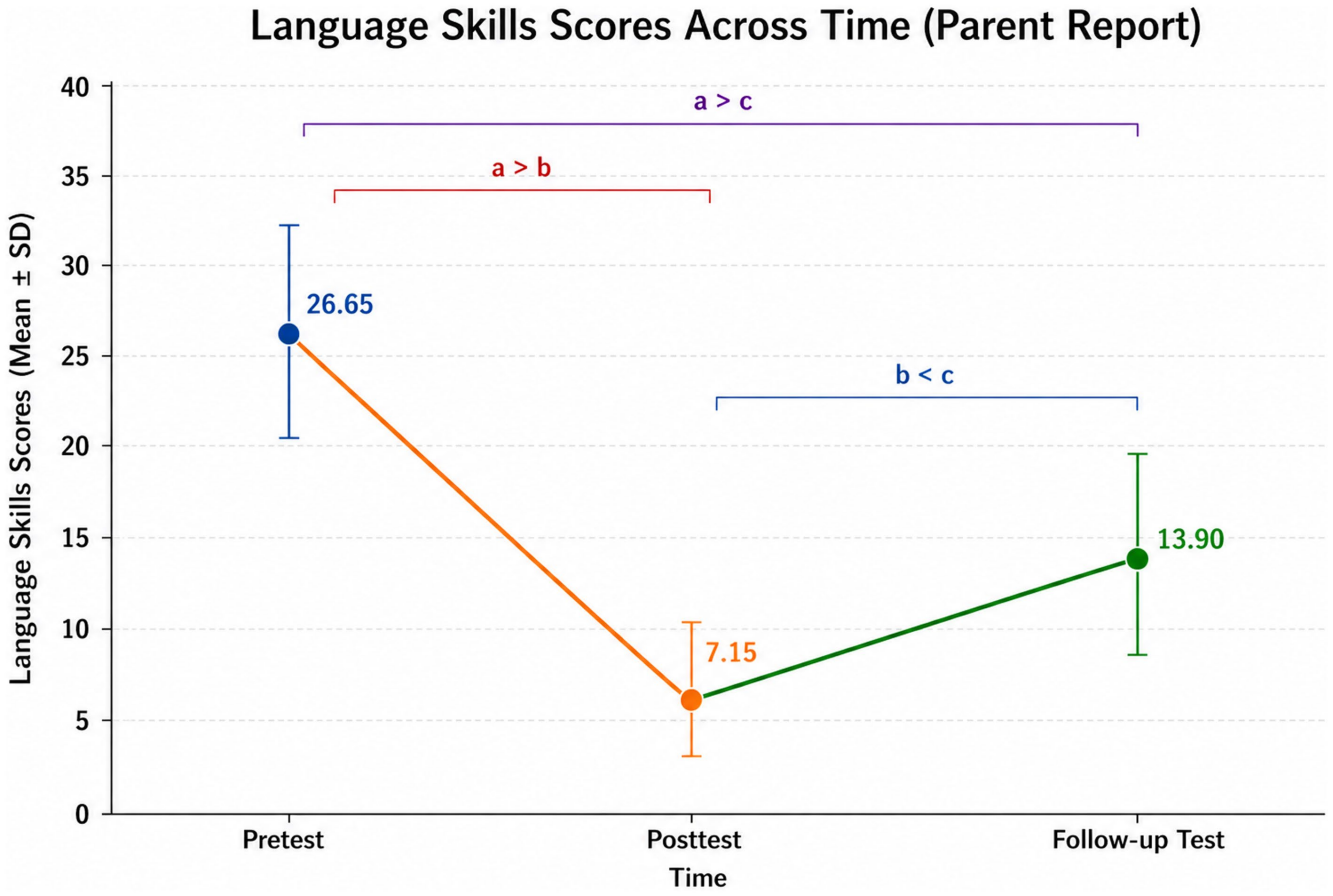
Change in language skills scores over time (Parent Report).

**Figure 5:**
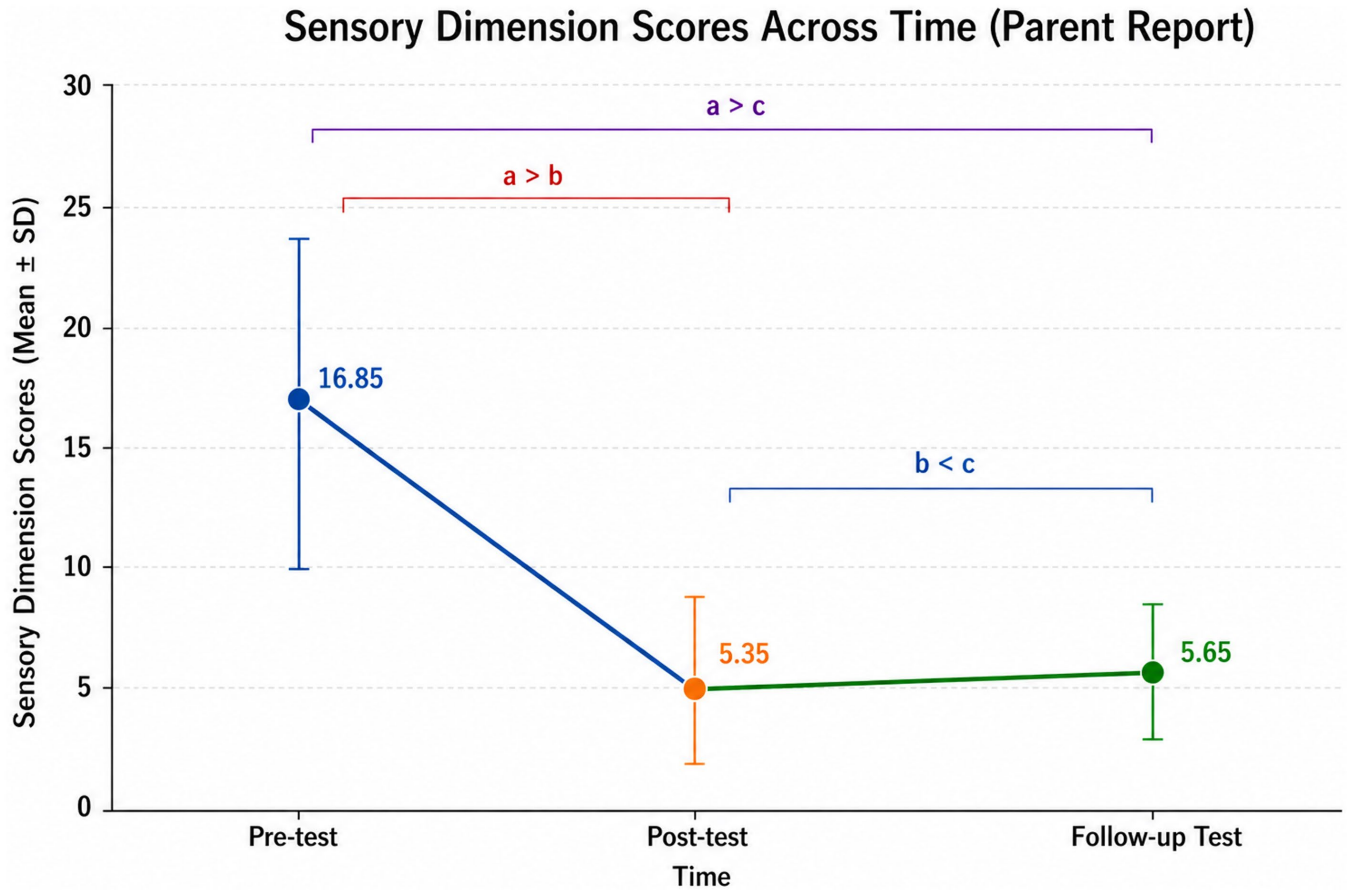
Sensory dimension scores over Time (Parent Report).

**Figure 6:**
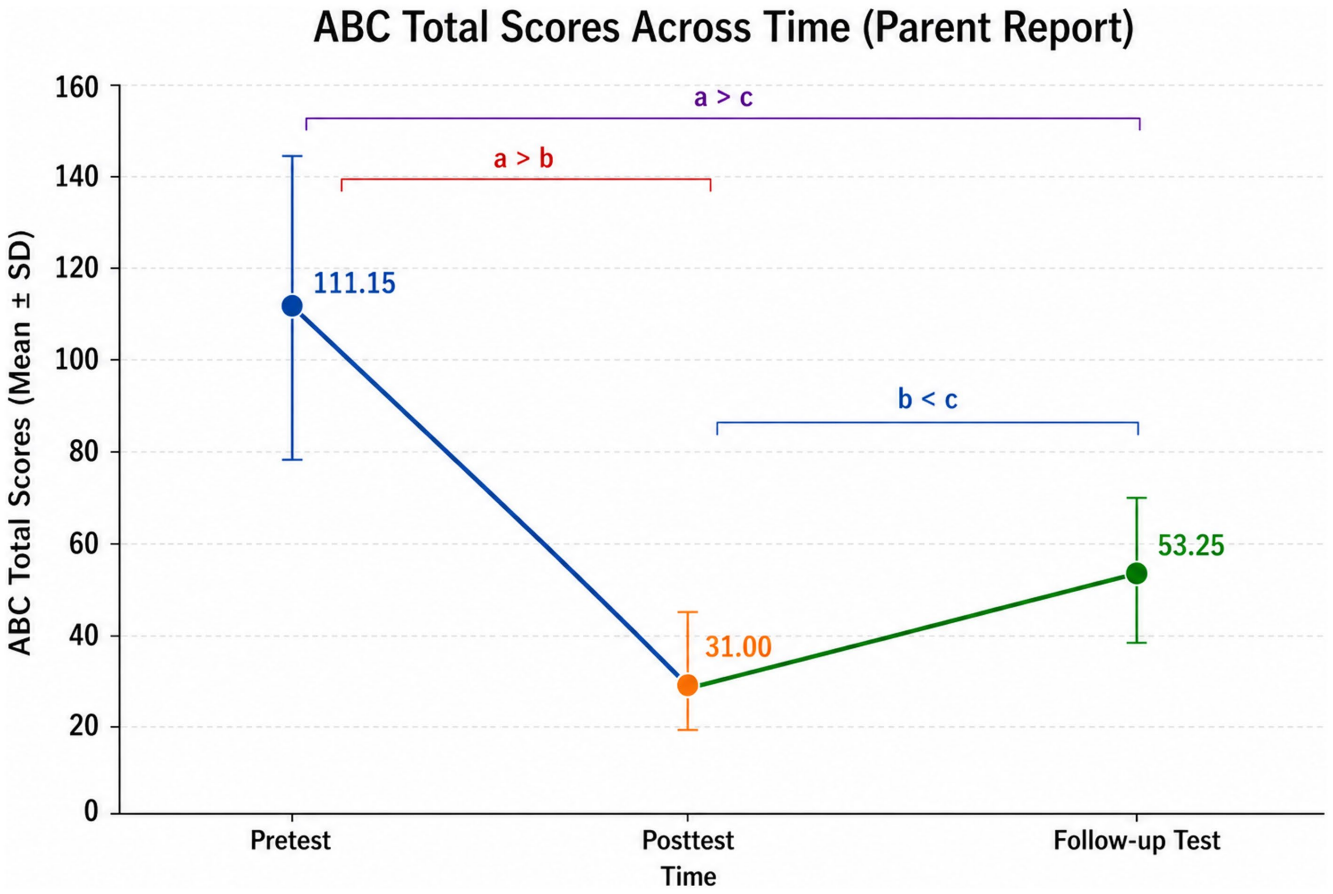
ABC total scores across time (Parent Report).

Therefore, the present study aims to address these gaps by examining the immediate and follow-up effects of a structured EAT program using parent-reported behavioral data (see Figures 1–6).

### 1.1 Theoretical framework: mechanisms underlying the effects of equine-assisted therapy in autism spectrum disorder

EAT may influence behavioral and functional outcomes in children with ASD through multiple, interrelated mechanisms. This theoretical framework integrates sensory integration theory, human–animal interaction theory, and social motivation theory to explain how engagement with the horse and the structured therapeutic context may support regulation, participation, and adaptive functioning.

From a sensory integration perspective, children with ASD commonly experience challenges in processing and organizing vestibular, proprioceptive, tactile, auditory, and visual inputs. The rhythmic and repetitive movement of the horse provides continuous multisensory stimulation that may support sensory modulation, postural control, and motor planning (Schaaf et al., 2014). Because equine-assisted activities require continuous bodily adaptation to the horse’s movement and surrounding environment, they may also promote embodied experiences that encourage greater awareness of movement and responsiveness to contextual demands in children with ASD (Peters et al., 2020).

Improvements in sensory regulation may facilitate more coordinated movement patterns and greater bodily awareness, which are essential for functional engagement in movement-based activities.

Human–animal interaction theory provides a complementary explanatory pathway. Interacting with animals may offer a non-judgmental, emotionally responsive context that supports emotional regulation and reduces anxiety. Recent intervention findings further suggest that emotionally supportive interactions with horses may reduce stress-related behaviors and improve task engagement among autistic children, potentially facilitating greater participation in therapeutic activities (Zhao et al., 2021; Peters et al., 2020). These emotional and relational processes may facilitate sustained engagement during therapy sessions.

Taken together, this framework suggests that EAT may influence children with ASD through interacting pathways involving sensory regulation, emotional engagement, and social participation. Accordingly, this framework provides the theoretical basis for interpreting behavioral changes observed following EAT in children with ASD.

Importantly, the mechanisms discussed in this section were not directly measured in the present study. Sensory regulation, emotional regulation, anxiety reduction, social motivation, and human–animal interaction pathways are presented as possible explanatory mechanisms based on previous literature, not as empirically tested mediators. Therefore, any interpretation concerning these mechanisms should be regarded as theoretical and speculative.

### 1.2 The present study

This study aimed to examine parent-reported behavioral changes following participation in a structured EAT program among children with ASD. The primary focus of the study was the assessment of behavioral changes across ABC domains over time.

Study Hypotheses

*H1*: Parent-reported ABC sensory dimension scores are expected to differ significantly across pretest, posttest, and follow-up measurements.

*H2*: Parent-reported ABC relationship-building scores are expected to differ significantly across pretest, posttest, and follow-up measurements.

*H3*: Parent-reported ABC body and object use scores are expected to differ significantly across pretest, posttest, and follow-up measurements.

*H4*: Parent-reported ABC social and self-care scores are expected to differ significantly across pretest, posttest, and follow-up measurements.

*H5*: Parent-reported ABC language skill scores are expected to differ significantly across pretest, posttest, and follow-up measurements.

*H6*: Parent-reported ABC total scores are expected to differ significantly across pretest, posttest, and follow-up measurements.

## 2 Methods

### 2.1 Study design

This study employed a longitudinal, parent-based, single-group design to examine changes in behavioral outcomes over time following participation in an 8-week EAT program in children diagnosed with ASD. The design was structured to explore temporal associations between the intervention and behavioral measures rather than to establish direct causal effects.

A total of 36 participants were assessed at three time points: pre-test (prior to the intervention), post-test (immediately after the intervention), and follow-up test (2 weeks after the completion of the program). Behavioral functioning was evaluated using the ABC, with data collected from parents through face-to-face administration under researcher supervision.

A longitudinal approach was selected because it enables the monitoring of developmental trends and the short-term continuity of intervention-related changes (Caruana et al., 2015). Parent-based reporting was preferred because parents are the most consistent observers of their children’s behavior across daily contexts, particularly in autism research (Matson and Nebel-Schwalm, 2007). Accordingly, the findings of the present study should be interpreted as indicative of behavioral change patterns associated with EAT participation over time.

Data collection was conducted in three stages. First, parents were informed about the study and completed the ABC scale to reflect their children’s baseline behavioral status. Second, children participated in the 8-week EAT program, which consisted of 2 sessions per week.

Following the intervention, parents completed the post-test assessment. In the final stage, children returned to their daily routines. Their parents observed them for an additional 2-week period, after which the ABC scale was re-administered as a follow-up measure to examine the short-term persistence of observed changes.

Given the single-group design, findings should be interpreted as within-group behavioral changes over time rather than causal intervention effects.

### 2.2 Determination of sample size

The sample size for this study was determined using G*Power 3.1 (Faul et al., 2007) via an *a priori* power analysis for a within-subjects repeated-measures ANOVA. Based on an anticipated large effect size (*f* = 0.40), an alpha level of 0.05, statistical power of 0.95, three measurements, and a correlation among repeated measures of 0.50 with nonsphericity correction set at 1, the analysis indicated that a minimum of 18 participants would be required to detect statistically significant differences. These parameters yielded a noncentrality parameter *λ* = 17.28, a critical *F* value = 3.28, and degrees of freedom of 2 and 34. The final sample size of 36 participants, therefore, exceeded the minimum requirement, providing adequate statistical power to detect meaningful within-group changes over time.

### 2.3 Research group

A purposive sampling strategy was employed due to the focus on a specific clinical population (Patton, 2014), and proximal (convenience) sampling was also used based on accessibility to the intervention setting (Yıldırım and Şimşek, 2021). Homogeneous sampling was preferred to ensure that participants shared similar diagnostic and functional characteristics, thereby enhancing internal consistency within the sample.

Children diagnosed with ASD according to the criteria of the Diagnostic and Statistical Manual of Mental Disorders constituted the target population of the study.

The study sample was recruited from children receiving services in private special education and rehabilitation centers located in various districts of Istanbul, including its diverse urban areas, where access to educational, therapeutic, and rehabilitative services is widely available. Istanbul was selected as the study setting not because of assumptions regarding ASD prevalence, but because it represents the primary geographical area in which the researchers have sustained professional access to special education and rehabilitation institutions, trained personnel, and EAT facilities, allowing the intervention and follow-up process to be conducted in a feasible and controlled manner.

Of 184 children aged 9–11 years receiving educational and rehabilitation services from four private special education and rehabilitation centers in socioeconomically and demographically comparable urban districts of Istanbul, 47 met the study criteria and were initially enrolled. Eleven children were excluded due to irregular attendance during the intervention period, resulting in a final sample of 36 participants (18 females and 18 males), with a mean age of 9.90 ± 1.44 years.

Participants were included in the study if they had a confirmed diagnosis of ASD according to DSM-5 criteria, demonstrated partial verbal communication skills, had not previously participated in any EAT program, this criterion was determined based on parent reports and routine functional information provided by the special education and rehabilitation centers, rather than through a separate standardized daily living skills assessment, were not receiving regular pharmacological treatment, and had no additional chronic medical condition or neurological disorder requiring ongoing medical treatment, apart from their ASD diagnosis. These criteria were applied to ensure a relatively homogeneous sample and to reduce the influence of confounding developmental or medical factors on behavioral outcomes.

Participants were excluded if they had any additional chronic disease, failed to attend two consecutive therapy sessions during the intervention period, or exceeded a body weight of 60 kg at any point during training. The 60 kg weight limit was implemented to ensure the safety of both the child and the therapy horse, in line with commonly accepted EAT safety and animal welfare principles, which emphasize matching rider weight to the horse’s physical capacity. This criterion was also consistent with the therapy center’s internal safety protocol and with international equine-assisted service principles emphasizing the protection of both participant safety and equine welfare (Horses in Education and Therapy International, 2023).

All parents were informed in detail about the study procedures, and written informed consent was obtained before participation.

To strengthen internal validity, all participants met strict inclusion criteria, had no prior exposure to EAT, did not receive concurrent pharmacological treatment, and were assessed using the same standardized instrument at all time points under researcher supervision. Accordingly, the study is reported in line with methodological guidelines for quasi-experimental and longitudinal intervention research rather than randomized controlled trial designs.

### 2.4 Autism behavior checklist (ABC)

ABC was developed by Krug et al. (1980), and its Turkish adaptation, validity, and reliability studies were conducted by Irmak et al. (2007). The ABC scale has five factors and 57 items. The scale factors include nine items for the sensory dimension, 12 for establishing relationships, 12 for body and object use, 13 for language skills, and 11 for social and self-care skills. The highest score possible on this scale is 159, and the lowest is 0. In other words, high scores on the scale indicate a high prevalence of autism symptoms. The ABC scale’s practical advantage is that it allows for information about children with autism to be obtained from educators and parents (Krug et al., 1980). In the Turkish validity-reliability study, the cutoff score was set at 39, and the correct classification rate was 88%. In reliability analyses, Cronbach’s alpha and split-half reliability were 0.92. Inter-rater reliability was 0.86 for 100 children assessed independently by two teachers and 0.59 for 69 children assessed by a teacher and a parent. Diagnosis and severity of the problem were used as external criteria to test criterion validity, and the results obtained supported the scale’s criterion validity. All these findings support the scale’s validity and reliability.

Although physical literacy was used as a conceptual framework in the present study, the ABC domains should not be interpreted as direct indicators of their components. Rather than assessing constructs such as physical competence, confidence, motivation, or participation, the ABC evaluates parent-reported behavioral characteristics associated with autism-related functioning. Therefore, physical literacy was considered only as a broader conceptual framework for interpreting behavioral findings, rather than as an outcome construct.

The use of the ABC in the present study was considered appropriate because the primary focus was the examination of parent-reported behavioral changes across domains such as sensory-related behaviors, communication, social interaction, and adaptive behavioral patterns. Furthermore, previous equine-assisted intervention studies involving children with ASD have frequently relied on caregiver-reported behavioral assessments to examine changes in ecologically meaningful contexts (Xiao et al., 2023). Accordingly, the present findings should be interpreted as behavioral outcomes assessed through parent reports rather than as indicators of broader developmental constructs.

### 2.5 Application of data collection tools (experimental procedure path)

Participating children received 480 min of training over 8 weeks, twice a week, for 16 sessions. Each session lasted 30 min. The therapy training for children with autism was conducted individually by a psychologist, a physiotherapist, a horse trainer, and two walker personnel. The training content applied is presented in Table 1.

**Table 1:** Equine-assisted therapy training plan.

Week	Day	Minute	Purpose / Goal	Event content
1	Day 1	30 min	Getting used to the environment and the horses	Introduction to the therapy center, observing horses, and practicing getting close to horses
	Day 2	30 min	First contact with the horse	Touching the horse with your hands and holding the horse’s mane while watching it
2	Day 1	30 min	Basic riding stances	Balance exercises by sitting on a horse, learning the correct sitting position
	Day 2	30 min	Movement awareness	Slight bending forward, backward, and sideways with the bust
3	Day 1	30 min	Upper body and arm coordination	Extending the hands forward, to the sides, and the feet, petting the horse
	Day 2	30 min	Lying on a horse	Lying forward and backward, on the back or face down
4	Day 1	30 min	Motor skills	Attempts to stand up with or without stepping on the stirrups
	Day 2	30 min	Object control	Games of throwing objects at targets with the right/left hand
5	Day 1	30 min	Social interaction	Communicating with the coach (greeting, saying your name)
	Day 2	30 min	Sensory awareness	Touching the horse’s front legs, noticing the mane, and hugging the horse
6	Day 1	30 min	Balance and direction	Trials of turning left and right, forward and backward, and steering a horse
	Day 2	30 min	Audiovisual matching	Saying the names of printed images and number recognition exercises
7	Day 1	30 min	Integration of mixed skills	Trials of rein holding, steering, and basic commanding
	Day 2	30 min	Mixed-task applications	Multitasking involving throwing objects, talking, and changing direction
8	Day 1	30 min	Skill reinforcement	Integrated repetition of all learned movements
	Day 2	30 min	Skill reinforcement	Integrated repetition of all learned movements
	—	—	Final observation and evaluation	Completing the Parent ABC form

The data collection process consisted of three phases.

1. Pretest phase: In this phase, the parents of the children included in the study were informed about the tests to be conducted and had them complete the ABC under the researcher’s supervision and with the parents’ active participation.
2. Training process and posttest phase: Before the training began, the participating children and their parents were interviewed and observed therapy horses to familiarize themselves with the therapy center and to help them adapt to the center. Participating children received horseback riding and training twice a week for 30 min per session for 8 weeks. The training included body and object use skills, relationship building skills, language skills, social and self-care skills, and developing sensory skills, including mounting and dismounting a horse; sitting correctly; holding and using the reins; bustling movements on the horse, forward-backward, side-to-side, and reaching movements with the hands; naming printed images shown to the child; reading numbers; throwing objects to targets with the right and left hands; developing dialogue with the trainer; standing up with and without stepping on the stirrups; sitting down and standing; reaching forward and backward and lying down on the horse; petting the horse with one’s hands; trying to touch the horse’s front legs with the right and left hands; and holding the horse’s mane. At the end of the training, the parents of the participating children completed the ABC scale under the researcher’s supervision. The training was conducted considering the age, gender, and housing status of the autistic children.
3. Testing the follow-up of the effect: Children who completed the therapy training were observed by their parents starting from the week the training ended, and after 2 weeks (in the 10th week), a follow-up test was conducted by having the parents fill out the ABC scale again under the supervision of the researcher.

### 2.6 8-week equine assisted therapy training

Duration: 8 Weeks (2 Days a Week / 30 Minutes a Day).

Approach: Easy to Difficult-Observation, Familiarization, Basic Skill Development, Motor Coordination, Communication, Social and Cognitive Development.

The decision to implement a uniform 8-week intervention protocol across all participants was made to ensure methodological standardization and to minimize variability in intervention delivery. Although individualized and adaptive practices typically characterize EAT, the present study adopted a fixed structure in order to control for differences in session content, intensity, and progression that could confound outcome interpretation. Standardization of the intervention allowed for a clearer examination of time-related changes in behavioral outcomes and improved internal consistency across participants. Furthermore, applying the same protocol to all children enabled systematic comparison of pre-, post-, and follow-up measurements within a single-group longitudinal design. This approach was considered particularly important in the absence of a control group, as it reduced the influence of therapist-driven variability and ensured that observed changes could be more confidently associated with exposure to the same intervention structure over time.

### 2.7 Statistical analysis

Data from the ABC pre-test, post-test, and follow-up tests were analyzed using IBM SPSS Statistics 25. Prior to inferential analysis, the distributional properties of the data were examined. Normality was assessed through histogram plots, skewness and kurtosis values, and the Shapiro–Wilk test. Based on these indicators, the data were found to meet the assumptions of normality (Büyüköztürk, 2007).

To evaluate changes in behavioral outcomes over time, a repeated-measures analysis of variance (ANOVA) was conducted. This approach allowed for the comparison of ABC scores across three measurement points: pre-test, post-test, and follow-up test. The analysis focused on identifying statistically significant differences over time and examining within-subject changes in behavioral development based on parental reports.

The assumption of sphericity was tested using Mauchly’s test. Where the assumption was met, the “Sphericity Assumed” values were considered in the interpretation of results. When the assumption of sphericity was violated, Greenhouse–Geisser corrections were applied to adjust the degrees of freedom.

To further explore the nature of significant differences between measurement points, pairwise comparisons were conducted using the Bonferroni correction method.

Effect sizes were calculated using Cohen’s d to determine the magnitude of observed differences. According to Cohen (2013), effect sizes were interpreted as small (*d* < 0.20), medium (*d* ≈ = 0.50), and large (*d* ≥ 0.80). In addition to Cohen’s d, partial eta squared (η^2^) values were reported for repeated measures ANOVA to indicate effect size.

In addition to statistical significance, effect sizes were evaluated for clinical relevance, particularly their practical implications for behavioral interventions in children with ASD.

Given that all data were collected from a single source (parent reports), the potential for common method bias was considered. Although the longitudinal design and repeated measurements may reduce this risk, future studies are recommended to incorporate multi-informant data and objective assessment methods to further minimize potential bias.

Table 2 is presented. Despite some significant Shapiro–Wilk tests, skewness and kurtosis were acceptable, and repeated-measures ANOVA is robust to moderate non-normality. Therefore, parametric tests were considered appropriate (Büyüköztürk, 2007). Given the small sample size, the robustness of repeated-measures ANOVA to moderate deviations from normality, and the acceptable skewness and kurtosis values, the use of parametric tests was supported.

**Table 2:** ABC scale skewness-kurtosis scores.

Sub-dimensions	Measurement	Skewness	Kurtosis	*p*
Body object use	Pre test	0.435	0.320	0.144
	Post test	0.578	0.937	0.002
	Follow-up test	0.446	−0.328	0.061
Social self care	Pretest	−0.250	0.860	0.001
	Post test	0.605	−0.844	0.008
	Follow-up test	0.566	−0.794	0.006
Relationship building	Pre test	0.225	−0.764	0.004
	Post test	0.954	−0.252	0.005
	Follow-up test	−0.207	0.121	0.057
Language skills	Pre test	−0.305	−0.547	0.012
	Post test	0.909	0.367	0.004
	Follow-up test	−0.291	0.078	0.053
Sensory dimension	Pre test	−0.466	−0.471	0.004
	Post test	−0.344	−0.691	0.066
	Follow-up test	0.940	0.364	0.088
ABC scale total	Pre test	−0.329	0.706	0.031
	Post test	0.810	−0.368	0.096
	Follow-up test	0.692	0.397	0.037

## 3 Results

In this section, data from the ABC were analyzed and presented in the tables below.

An examination of Table 3 revealed a significant difference in the body object use subscale between the pretest, posttest, and follow-up test scores, according to parents’ opinions (*p* < 0.01). The Bonferroni test showed significant differences between the pretest–posttest, pretest–retest, and posttest–retest. The within-group effect size was η^2^ = 0.576, indicating a significant effect according to Cohen’s classification. The time effect (within-subjects effect) yielded η^2^ = 0.633, indicating a large effect (Cohen, 2013).

**Table 3:** Repeated measures ANOVA results for pre-test, post-test, and follow-up test values for body object use level.

Variable	Sample	*N*	Pre test^a^	Post test^b^	Follow-up test^c^	F	*p	η^2^	Bonferroni
			X ± Ss	X ± Ss	X ± Ss				
Body object use	Parent	36	23.95 ± 9.71	8.50 ± 2.83	12.40 ± 7.30	22.730	0.000	0.576	a > b a > c b < c
	**F: 43.197, *p*: 0.000, η^2^: 0.633								

Mauchly’s Test of Sphericity *p* = 0.059; *Within-group comparison (Tests of within-subjects effects, pre-, post- and follow-up test), ** Tests of Within-subject effects across time; *p* < 0.01.

When Table 4 is examined, a significant difference was found between the pretest, posttest, and follow-up test scores in the social self-care level subscale according to parents’ opinions (*p* < 0.01). The Bonferroni test showed that there were differences between all measurements (a > b, a > c, b < c). The within-group effect size was η^2^ = 0.717, and the time effect (within-subject effect) was η^2^ = 0.777, both values being large according to Cohen’s classification (Cohen, 2013).

**Table 4:** Repeated measures ANOVA results for pre-test, post-test, and follow-up test values of social self-care level.

Variable	Sample	*N*	Pre test^a^	Post test^b^	Follow-up test^c^	F	*p	η^2^	Bonferroni
			X ± Ss	X ± Ss	X ± Ss				
Social self-care	Parent	36	17.90 ± 5.94	3.65 ± 3.21	8.80 ± 5.53	48.059	0.000	0.717	a > b a > c b < c
	**F: 66.137, *p*: 0.000, η^2^: 0.777								

Mauchly’s Test of Sphericity *p* = 0.113; *Within-group comparison (Tests of within-subjects effects, pre-, post-, and follow-up test), ** Tests of Within-subject effects across time; *p* < 0.01.

An examination of Table 5 revealed a significant difference in the relationship-building subscale between pretest, posttest, and follow-up test scores, according to parental opinions (*p* < 0.01). The Bonferroni test showed differences across all measurements (a > b, a > c, b > c). According to Cohen’s classification, the within-group effect size was η^2^ = 0.696, indicating a large effect size. The time effect (within-subjects) was η^2^ = 0.883, indicating a large effect size (Cohen, 2013).

**Table 5:** Repeated measures ANOVA Results for pre-test, post-test, and follow-up test values of the level of establishing relationships.

Variable	Sample	*N*	Pre test^a^	Post test^b^	Follow-up test^c^	F	*p	η^2^	Bonferroni
			X ± Ss	X ± Ss	X ± Ss				
Relationship building	Parent	36	25.80 ± 9.36	6.35 ± 3.23	12.50 ± 4.45	43.522	0.000	0.696	a > b a > c b < c
	**F: 142.924, *p*: 0.000, η^2^: 0.883								

Mauchly’s Test of Sphericity *p* = 0.077; *Within-group comparison (Tests of within-subjects effects, pre-, post-, and follow-up test), ** Tests of Within-subject effects across time; *p* < 0.01.

When Table 6 was examined, a significant difference was found between the pretest, posttest, and follow-up test scores in the language skill level subscale according to parents’ opinions (*p* < 0.01). The Bonferroni test showed that there were differences between all measurements (a > b, a > c, b < c). According to Cohen’s classification, the within-group effect size was η^2^ = 0.727, indicating a large effect. The time effect (within-subjects) was η^2^ = 0.954, indicating a very large effect size (Cohen, 2013).

**Table 6:** Repeated measures ANOVA results for pre-test, post-test, and follow-up test values of language skill level.

Variable	Sample	*N*	Pretest^a^	Posttest^b^	Follow-up test^c^	F	*p	η^2^	Bonferroni
			X ± Ss	X ± Ss	X ± Ss				
Language skills	Parent	36	26.65 ± 6.10	7.15 ± 3.01	13.90 ± 5.43	50.670	0.000	0.727	a > b a > c b < c
	**F: 392.612, *p*: 0.000, η^2^: 0.954								

Mauchly’s Test of Sphericity *p* = 0.071; *Within-group comparison (Tests of within-subjects effects, pre-, post-, and follow-up test), **Tests of Within-subject effects across time; *p* < 0.01.

When Table 7 was examined, a significant difference was found between the pretest, posttest, and follow-up test scores at the sensory dimension according to parents’ opinions (*p* < 0.01). The Bonferroni test showed that there were differences between all measurements (a > b, a > c, b < c). According to Cohen’s classification, the within-group effect size was η^2^ = 0.616, indicating a large effect. The time effect (within-subject effect) was η^2^ = 0.932, corresponding to a very large effect size (Cohen, 2013).

**Table 7:** Repeated measures ANOVA results for pre-test, post-test, and follow-up test values of sensory dimension.

Variable	Sample	*N*	Pre test^a^	Post test^b^	Follow-up test^c^	F	*p	η^2^	Bonferroni
			X ± Ss	X ± Ss	X ± Ss				
Sensory dimension	Parent	36	16.85 ± 6.80	5.35 ± 3.39	5.65 ± 2.43	30.493	0.000	0.616	a > b a > c b < c
	**F: 260.702, *p*: 0.000, η^2^: 0.932								

Mauchly’s Test of Sphericity *p* = 0.101; *Within-group comparison (Tests of within-subjects effects, pre-, post-, and follow-up test), **Tests of Within-subject effects across time; *p* < 0.01.

When Table 8 is examined, a significant difference was found between the pretest, posttest, and follow-up test scores in the ABC total score according to parents’ opinions (p < 0.01). The Bonferroni test showed that there were differences between all measurements (a > b, a > c, b < c). According to Cohen’s classification, the within-group effect size was η^2^ = 0.719, indicating a large effect size. The time effect (within-subjects) was η^2^ = 0.964, indicating a very large effect size (Cohen, 2013).

**Table 8:** Analysis of pre-test, post-test, and follow-up test values of the ABC total level.

Variable	Sample	*N*	Pretest^a^	Posttest^b^	Follow-up test^c^	F	*p	η^2^	Bonferroni
			X ± Ss	X ± Ss	X ± Ss				
ABC total	Parent	36	111.15 ± 34.65	31.0 ± 12.01	53.25 ± 14.98	48.657	0.000	0.719	a > b a > c b < c
	**F: 506.261, *p*: 0.000, η^2^: 0.964								

Mauchly’s Test of Sphericity *p* = 0.097; *Within-group comparison (Tests of within-subjects effects, pre-, post- and follow-up test), ** Tests of Within-subject effects across time; *p* < 0.01.

The results indicate significant changes in parent-reported ABC scores across measurement points. Specifically, decreases in ABC subscale and total scores suggest reductions in parent-reported autism-related behavioral symptoms from pretest to posttest, with some changes partially maintained at follow-up. Given the single-group design, these findings should be interpreted as within-group behavioral change patterns over time rather than causal intervention effects.

## 4 Discussion

The present study examined changes in parent-reported behavioral outcomes following participation in an 8-week EAT program among children with ASD using the Autism Behavior Checklist (ABC). Given the longitudinal single-group design and the reliance on parent-reported assessments, the findings should be interpreted as patterns of within-group behavioral change over time rather than evidence of causal intervention effects. To improve conceptual clarity, the findings are discussed according to the behavioral domains directly assessed by the ABC.

### 4.1 Sensory dimension

The findings demonstrated significant changes in parent-reported sensory-related behaviors across measurement points.

One possible interpretation of these findings is that repeated exposure to horse-related movement experiences may have provided structured opportunities for sensory adaptation and environmental responsiveness. The rhythmic, multidirectional movement of the horse may require continuous vestibular and proprioceptive adjustment, potentially contributing to observable changes in sensory-related behavioral patterns reported by parents. Importantly, this interpretation may suggest a participation-related mechanism rather than direct sensory rehabilitation.

These findings may also contribute to the literature by extending prior intervention studies through the inclusion of a follow-up assessment. While previous studies primarily focused on immediate post-intervention outcomes, the present findings provide preliminary evidence regarding the short-term maintenance of parent-reported behavioral changes over time (Borgi et al., 2016; Gabriels et al., 2012).

Similar patterns have been reported in previous equine-assisted intervention studies demonstrating improvements in sensory responsiveness and behavioral regulation among children with ASD (Zhao et al., 2021; Peters et al., 2020; Xiao et al., 2023). Previous therapeutic riding research has further suggested that vestibular and proprioceptive stimulation may represent potentially relevant processes associated with behavioral organization in autistic children (Zhao et al., 2021). Consistent with these observations, other equine-assisted intervention studies have proposed that the rhythmic movement of the horse may provide multisensory experiences associated with sensory responsiveness and adaptive behavioral functioning among children with ASD (Ward et al., 2013; Gabriels et al., 2012; Bass et al., 2009).

However, these interpretations should be approached cautiously. Sensory processing itself was not directly assessed in the present study, and ABC sensory items reflect observable parent-reported behaviors rather than underlying sensory mechanisms. Therefore, sensory integration pathways should be regarded only as possible theoretical explanations rather than empirically tested mechanisms.

### 4.2 Relationship-building and language skills

The present findings also demonstrated changes in parent-reported relationship-building and language-related behaviors. One potential interpretation is that EAT sessions may create opportunities for interaction through therapist-guided activities, turn-taking, and horse-related communication tasks embedded in meaningful contexts. Such structured experiences may provide repeated opportunities for social engagement and communication-related behaviors.

Previous literature similarly suggests that equine-assisted programs may be associated with improvements in social responsiveness, communication-related behaviors, and engagement among children with ASD (Peters et al., 2020; Zhao et al., 2021; Sylvia et al., 2024). Related intervention studies have also reported improvements in social communication and engagement in structured equine-assisted settings involving interaction with therapists and animals (Lanning et al., 2014; Gabriels et al., 2012; Bass et al., 2009).

Beyond consistency with previous findings, the present study contributes to the literature by examining whether parent-reported behavioral changes appeared to persist beyond the intervention period. Unlike many previous equine-assisted studies that focused exclusively on immediate post-intervention findings, the inclusion of follow-up assessment provides preliminary evidence regarding the temporal continuity of communication-related behavioral changes.

Nevertheless, these explanations remain speculative because social motivation, communication processes, and mechanisms of emotional regulation were not directly measured in the present study. Therefore, these interpretations should be considered theoretical rather than empirically verified pathways.

### 4.3 Body and object use

Significant changes were also observed in parent-reported body- and object-use behaviors.

One interpretation is that equine-assisted activities expose children to repeated balance adjustments and movement coordination demands within meaningful interaction contexts. Such experiences may create opportunities for engagement that differ from conventional therapeutic exercises because movement, environmental feedback, and social interaction occur simultaneously.

Previous studies have suggested that equine-assisted activities involve balance adjustments, coordinated movement patterns, and continuous postural adaptation, which may be relevant to behavioral and functional development in children with ASD (Peters et al., 2020; Xiao et al., 2023).

Previous intervention findings additionally suggest that therapeutic riding environments may create opportunities for motor coordination, balance control, and postural engagement through repeated interaction with dynamic movement conditions (Akpınar et al., 2016; Özyurt et al., 2017; Llambias et al., 2016).

The structured and task-oriented nature of EAT sessions may provide repeated opportunities for coordinated movement and environmental responsiveness. However, developmental interpretations involving processes such as postural regulation, motor coordination, or developmental cascade pathways remain theoretical. Because these mechanisms were not directly assessed, it cannot be determined whether observed behavioral patterns reflect specific developmental processes or broader maturational changes occurring over time.

### 4.4 Social and self-care skills

Changes observed in parent-reported social and self-care behaviors may suggest that repeated participation in structured therapeutic routines created opportunities for behavioral engagement beyond isolated skill practice. Activities involving interaction with therapists, horses, and environmental tasks may have promoted repeated exposure to social participation experiences. These findings may indicate that behavioral changes occurred within meaningful activity contexts rather than solely through isolated therapeutic exercises.

Prior studies similarly suggest that structured equine-assisted activities may provide opportunities for guided participation and repeated social interaction (Peters et al., 2020; Zhao et al., 2021). Additional findings suggest that equine-assisted contexts may encourage participation through therapist guidance, shared task completion, and meaningful interaction opportunities (Ward et al., 2013; Borgi et al., 2016).

However, because daily functioning, adaptive participation, and independence were not directly measured, interpretations extending beyond ABC scores should be avoided. Therefore, findings should remain limited to observed changes in parent-reported behavioral domains rather than broader conclusions regarding functional development.

Taken together, reductions across several ABC domains suggest a broader pattern of parent-reported behavioral change following EAT participation; however, these findings should be interpreted cautiously and should not be considered evidence of generalized developmental effects.

### 4.5 Physical literacy as a conceptual framework

Physical literacy was introduced in the present study solely as a conceptual framework to contextualize the findings, not as an outcome variable. Importantly, physical literacy was not directly assessed using a standardized or validated instrument. Therefore, the findings should not be interpreted as evidence of physical literacy development.

Recent literature has suggested that several constructs frequently discussed within animal-assisted intervention research such as movement engagement, confidence, and participation may conceptually overlap with dimensions commonly associated with physical literacy (Sylvia et al., 2024). Nevertheless, these conceptual similarities remain indirect and should not be interpreted as empirical evidence. Rather than indicating physical literacy development, the present findings may simply suggest directions for future research incorporating validated physical literacy measures.

### 4.6 Limitations and future directions

Several limitations should be considered when interpreting the findings of the present study. First, the study relied exclusively on parent-reported measures using the ABC. Although caregiver reports provide ecologically meaningful information regarding children’s behaviors across everyday contexts, such assessments may also introduce expectancy effects, subjective interpretation, and reporting bias. Consequently, the absence of multi-informant approaches, including teacher reports, clinician assessments, or direct behavioral observations, reduces the objectivity of outcome evaluation. Second, the absence of a control group substantially limits causal interpretation and makes it difficult to determine whether observed behavioral changes were specifically associated with EAT participation. Given the single-group longitudinal design, alternative explanations including maturation, regression to the mean, novelty effects, increased adult attention, concurrent therapies, educational experiences, family involvement, physical activity participation outside the intervention setting, and broader environmental influences may also have contributed to the observed findings.

Third, the relatively small sample size and inclusion of only children with partial verbal communication abilities restrict generalizability. Although the sample was sufficient for detecting within-group changes, it may not adequately represent the broader ASD population, particularly individuals with varying severity levels, non-verbal profiles, or comorbid developmental conditions. Future studies should therefore include larger and more diverse samples involving different age groups, varying ASD severity levels, non-verbal profiles, and individuals with comorbid developmental conditions to provide a broader understanding of intervention applicability.

Fourth, the follow-up period was limited to 2 weeks, preventing conclusions regarding the long-term maintenance and generalization of behavioral changes. Accordingly, future longitudinal studies incorporating extended follow-up assessments (e.g., 3-, 6-, or 12-month periods) are needed to determine whether observed behavioral changes persist over time and generalize across home, school, and community settings.

Finally, although statistically significant findings and large effect sizes were observed, clinical significance was not formally evaluated using standardized benchmarks or minimal clinically important difference (MCID) criteria. In addition, potential explanatory mechanisms discussed throughout the present study including sensory regulation, emotional responsiveness, social motivation, and human–animal interaction pathways were not directly assessed and therefore remain theoretical.

Future studies should employ randomized controlled designs incorporating appropriate comparison conditions such as waitlist controls, treatment-as-usual groups, or alternative activity interventions to determine whether observed behavioral changes are specific to EAT participation or reflect broader contextual influences. Multi-informant and multi-method assessment approaches should also be adopted by incorporating teacher reports, clinician assessments, standardized behavioral observations, and independent evaluators. Inclusion of objective indicators such as physiological measures (e.g., heart rate variability and cortisol levels) or movement-based technologies may further strengthen methodological rigor. Future studies should additionally investigate potential mechanisms underlying EAT-related behavioral changes through mediation analyses and experimentally controlled designs. Further refinement and standardization of intervention protocols, including comparisons of intervention duration, session frequency, therapist characteristics, horse-related variables, and environmental conditions, may also contribute to identifying optimal implementation strategies. Finally, future research should integrate both statistical and clinical indicators, including MCID benchmarks and qualitative perspectives from parents and children, to better distinguish statistically significant findings from clinically meaningful outcomes.

## 5 Conclusion

In conclusion, the present study found that participation in an 8-week EAT program was associated with changes in parent-reported behavioral outcomes, as assessed using the ABC, among children with ASD. Specifically, changes were observed across several ABC subdomains, suggesting that participation in the program coincided with variations in parent-reported behavioral patterns over the intervention period. Although some changes appeared to be partially maintained at short-term follow-up, these findings should be interpreted cautiously because the study employed a single-group longitudinal design without a control condition. Therefore, the results should be interpreted as within-group behavioral change patterns over time rather than evidence of causal intervention effects, and it cannot be determined whether the observed changes were attributable specifically to EAT participation.

Given these methodological considerations, the present findings should be regarded as preliminary evidence supporting the feasibility of EAT as a complementary intervention approach rather than as evidence of clinical efficacy. Future studies employing randomized controlled designs, larger sample sizes, extended follow-up periods, and multidimensional assessment approaches are needed to clarify the robustness, sustainability, and specificity of behavioral outcomes associated with EAT participation.

Importantly, although the intervention involved movement-based activities and structured engagement experiences, physical literacy was not directly assessed using a standardized instrument and therefore cannot be interpreted as an outcome of the present study. Any conceptual links between EAT participation and broader participation-related constructs should remain tentative and should be examined in future research using validated and comprehensive assessment tools.

Overall, the present study contributes to the existing literature by providing parent-reported behavioral data from children with ASD participating in a structured EAT program and highlights the need for more methodologically rigorous research to better understand the scope, specificity, and potential mechanisms associated with equine-assisted interventions within multidisciplinary support frameworks.

## Data Availability

The raw data supporting the conclusions of this article will be made available by the authors, without undue reservation.
