# Supplementary material for: Parent-reported behavioral changes following equine-assisted therapy in children with autism spectrum disorder: a single-group longitudinal study
Source: Front Psychol. 2026 Jun 5;17:1869472. doi: 10.3389/fpsyg.2026.1869472 (PMC13279089; doi:10.3389/fpsyg.2026.1869472)
Supplement: Supplementary file 1 [file Supplementary_file_1.pdf]

frontiers

Appendix A. EAT Protocol for Children with Autism Spectrum Disorder

Objectives

Sensory Development: Reducing children's sensory sensitivity to environmental stimuli and increasing their sensory awareness.

Communication Skills: Developing verbal and nonverbal communication skills.

Body and Object Use: Developing children's body awareness, balance, coordination, and motor skills.

Social and Self-Care Skills: Increasing social interaction and developing basic self-care skills (eating, dressing, etc.).

Emotional and Psychosocial Development: Providing opportunities that may support self-confidence and managing their emotional reactions.

Session Preparation and General Structure

Duration: Two sessions per week, each 30 minutes long.

Session Setting: Large areas where children feel comfortable and safe and can sustain their attention (horse arena, safe exercise areas).

Participation Requirements: Children must wear comfortable clothing, appropriate footwear, and pre-therapy relaxation exercises (providing a safe environment).

Therapeutic Areas and Application Methods

A. Sensory Development

Purpose: To increase the child's sensory awareness of environmental stimuli.

Method

Children's sensory adaptation to body movement is supported through movements at different speeds on or around the horse's back.

Tolerance to sensory stimuli is increased by horseback riding on different surfaces (soft, hard) and environmental conditions (windy, sunny, dark).

Children's tactile awareness is enhanced by touching different parts of the horse (the mane, back, belly, etc.) during therapy.

B. Developing Communication Skills

Purpose: To develop the child's communication skills.

Method

During horseback rides, children are encouraged to use basic commands like "stop," "start," "slow," and "fast" to strengthen their communication skills.

Nonverbal communication is emphasized, helping children understand the relationship between the horse's movements and their own body language.

Non-verbal communication, such as eye contact, facial expressions, and gestures, is emphasized.

C. Body and Object Use

Purpose: To develop children's body awareness and motor skills.

Method

Body balance and coordination are trained during horseback riding exercises.

Body awareness improves through horseback riding in different positions (standing, sitting, on the back, etc.).

Balance exercises are initiated on the horse. They are taught the skills to overcome the fear of falling and to move safely.

D. Developing Social and Self-Care Skills

Purpose: To develop the child's social interaction skills and increase self-care abilities.

Method

Interaction between the therapist and the child is enhanced during horseback riding. Social interaction is encouraged as the therapist teaches the child how to manage the horse.

The therapist helps the child develop a sense of responsibility by engaging in basic self-care activities (e.g., cleaning, feeding, and grooming the horse) before and after riding.

Group activities (such as shared horseback riding with other children) can be organized to support the child's healthy communication with others.

E. Emotional and Psychosocial Development

Purpose: To increase the child's self-confidence and provide opportunities related to emotional engagement and self-expression.

Method

Children are given freedom during horseback riding, helping them learn about themselves and feel safe.

During the sessions, children are encouraged to love and trust the horse, strengthening emotional bonds.

Children are encouraged to confront their fears, relax, and focus on one thing at a time.

Session Structure and Content

Each session may follow the following structure:

Warm-up and Preparation (5 min)

Light stretching exercises to help children relax and prepare for the session.

Brief eye contact and trust-building exercises next to the horse.

Main Therapy Applications (20 min)

Horseback walks and rides at different speeds.

Developing communication skills by teaching children basic commands.

Motor skill exercises: balance exercises, guiding the horse.

Social interaction, games, and group activities.

Self-care activities: grooming and cleaning the horse.

Cool Down and Evaluation (5 min)

Relax and sit in a safe area after the final horseback ride.

Brief conversation about the child's feelings.

Brief family feedback, communication with parents about the child's development.

Monitoring and Evaluation

Assessment Tools

Children's developmental levels are monitored using scales such as the Autism Behavior Checklist (ABC).

Behavioral development is assessed through brief observations at the beginning and end of sessions.

Regular feedback meetings are held with parents.

Tracking and Adaptation

The child's progress is monitored, and session structures are modified if necessary.

Therapeutic goals are reviewed every 4-6 weeks based on the child's development.
